# Case of Fatal Colic from the Lodgement of a Chocolate Nut in the Appendicula Vermiformis

**Published:** 1816-02

**Authors:** Oliver Prescott

**Affiliations:** Newburyport.


*For the London Medical and Physical Journal.*

### *Case of Fatal Colic from the Lodgement of a Chocolate Nut in the Appendicula Vermiformis;* by Oliver Prescott, A.M. of Newburyport.

AS I do not recollect meeting with any case on record similar to the following, and as it is very possible that an extraneous substance, such as plum or cherry stones, may have in other instances given rise to similar cases, and thus have proved the hidden and unsuspected cause of obstinate and incurable colic;* I have thought it might be useful to make a communication of it to the public.

On the afternoon of Tuesday, the 10th of January, 1815, I was requested to visit Captain Parker Robert, of this town, aged forty-two years; he has for some years past been master of a merchant-vessel in the European trade; his health has always been good, never having experienced any sickness or considerable indisposition in his life, or had occasion (as he says) to apply for medical aid, excepting in one instance, which was at Cadiz about twelve months since, as will be mentioned. His complaint was an obtuse, deep-seated pain in the right side of the abdomen, just above the os ilium, but towards the lumbar region, accompanied by a tenderness of the bowels generally. He has experienced these sensations constantly, more or less, for several days; and expecting that he should find relief by an evacuation of his bowels, he had taken of his own accord, two days previous to my seeing him, a dose of rhubarb, which he says operated freely, but did not afford the expected relief, or produce any alleviation of the pain. His eyes had a yellow tinge, and his countenance exhibited a jaundiced hue, but there was no hardness or acceleration of his pulse, or morbid heat of the surface; his tongue also was clean. From the part pointed out, as the more immediate seat of pain, I concluded its source to be in the cœcum, or commencement of the colon, occasioned by a deficiency of bile in the intestines, and probably an accumulation of indurated fœces.

I prescribed a few grains of calomel, and directed them to be followed in half an hour by a dose of pills composed of aloes g. gamb. and nitre. When I put the pills into his

* About three years ago, a plum-stone swallowed proved fatal to the daughter of an eminent practitioner in Southampton-row, London. The subject was examined after death, and inflammation found in every part of the abdominal viscera.—Edit.

hand, hand, he enquired if they were bilious pills, saying that he had once experienced a similar affection of the bowels, about a year since, when at Cadiz; that he applied at that time to an English physician for advice, who gave him for the complaint a few pills, which he called bilious pills— these operated well, and afforded so much relief that no other medicine was taken, but that several days elapsed before his bowels felt entirely free from tenderness.

He took but four pills, which produced four copious ejections, and each operation was followed by mitigation of his pain. About the middle of the following night, he complained of feeling cold, and had a severe ague fit, his wife applied a warm woollen-blanket next to his body, and passed the warming-pan about him. As the chill went off, the pain returned with increased violence, and soon became so severe that I was sent for. I found him in very great distress, the pain being fixed altogether in the place of its origin, viz. above the spine of the right ilium, and as I supposed at the commencement of the colon. His pulse was small, but slow and soft; and as the cathartic had operated so freely, the pain was now attributed to spasm. A dose of opium was accordingly prescribed and repeated two or three times; the whole quantity given might have been eight or nine grains in all, after which he became easy and disposed to sleep; but this calm, however, was only of short duration, for in about half an hour he aroused in very great agony—but his distress was now of a different kind, his sensations being as if his bowels were distended to the utmost, so that there could be no room for him to breathe; he felt also a pressing inclination to pass his urine, and said repeatedly that his bladder would burst if his urine did not soon flow. His abdomen on examination was found rather tense, but not greatly swelled or inflated, and there was no such tumor above the pubes as is usual where much urine is collected in the *vesica urinaria.* Dr. Bradstreet was called in at this time, and concluded with me in consultation, that these symptoms were occasioned altogether by spasm. Camphorated oil was rubbed on the abdomen, a large epispastic applied over the original seat of pain, and an enema administered, which was also soon repeated. In an hour or two he found considerable relief, but without having passed any urine; during this distress preparation was made for drawing of blood, but the coldness of the surface and smallness of the pulse deterred us from opening a vein. Eight grains of calomel with camphor and nitre were now prescribed, with directions to repeat the like dose every six hours; he was also directed to take frequently of supercarbonate of potass dissolved dissolved in water, in the proportion of one drachm of the former to two pounds of the latter. I visited him at evening and found he had passed a more comfortable day than was expected, and his urine had passed without much difficulty; he was directed to continue the same course of medicines, and to take two grains of opium if the pain should become severe.

12th, Thursday. He has had a night tolerably free from pain, but has taken opium at different times to the amount of six grains in all—perspiration is free and has been so through the night—the pain has shifted and is now most severe on the opposite side of the abdomen. The epispastic was directed to be applied over the part now in most pain, and the patient ordered to continue the powders and follow the other directions. In the evening Dr. Noyes visited him with me, and advised a continuance of the same course of medicines, &c. and as there had been but two very small discharges from the bowels this day, or even since the morning of the 11th, it was agreed that he should take two ounces of ol. ricini early the next morning: his pulse is full and soft.

13th, Friday. Visited this morning with Dr. Noyes—he has taken but one grain of opium and has had considerable rest during the last night—the symptoms and his appearance are such as encourage the hope of a favourable termination of the disease—the powders of calomel, nitre and camphor, and the solution of subcarbonate of potass were advised to be continued, and he was directed to take this afternoon one ounce of manna and one fourth of an ounce of senna in infusion—the like dose to be repeated every hour, until there is a free operation from the bowels.

When I visited him at evening, he had taken three doses of the manna and senna, and after trying to introduce an injection, had a pretty copious discharge from his bowels whilst I was present. I would observe that a discharge from the bowels always afforded temporary relief; he therefore was often pressing to have an enema injected. In the night he became extremely sick at the stomach, and puked several times—I was sent for, and after applying appropriate remedies the nausea wore off.

14th, Saturday. I found him at the morning visit tolerably easy, but he had had no stool since the one in the evening—I prescribed a pill containing two grains of aloes to be taken every second hour, and every other hour two grains of calomel, and that he should take two grains of extract of hyosciamus whenever the pain should prove severe. In the evening his abdomen was much less tumid and he had less pain, but there had been no alvine discharge—he was therefore directed rected to continue the pills, but to increase the dose of calomel to three grains—the camphor, &c. to be continued.

15th, Sunday. I was sent for at nine o'clock A. M. He has enjoyed a comfortable night, with frequent naps of sleep, &c. but he now feels again that sensation of fulness and distention as if bursting—there has been no discharge from his bowels since the evening of the 13th, although numerous injections have been administered. He was now directed to take one drachm of manna in a solution of cream of tartar, and repeat the dose every half hour—three ounces were thus administered in the space of one hour and an half; it produced no operation, and the distressing sensation of his bowels being ready to burst asunder increased. A blister was applied over the stomach and bowels, with such other directions as were deemed most appropriate to the circumstances of the case. At two o'clock P. M. Dr. Noyes visited him with me—there has been no operation from the cathartic medicines, and injections cannot now be received or retained—his pulse is accelerated to one hundred and forty beats in a minute, and are so small as to be felt or counted but with difficulty—his extremities are cold, and his breathing very laborious—his countenance is also changed, and he is evidently dying—he lived until about nine o'clock in the evening and then expired.

Permission having been obtained, his body was opened and inspected the day after his decease, by Dr. Noyes and myself. Inflammation had universally pervaded the abdominal contents, the intestines were agglutinated together and covered with coagulated lymph, and were found adhering in many places to the peritonæum ; the cœcum and a considerable piece of the colon in its vicinity, as also part of the ileum, was in a complete state of sphacelation, and a small hard substance protruded through the cœcum, at the entrance of the appendix vermiformis, or rather at the mouth of the appendicula. This substance at first view was taken for a calculous concretion, but on applying the point of the scissars a piece of it broke off, and proved to be a cocoa or chocolate nut ; this had been lodged in the entrance of the appendix, and no doubt was the immediate cause of the patient's death ; and probably the same kind of complaint, mentioned as having happened at Cadiz a twelvemonth previous, was occasioned by this same nut. There were no scybala or hardened fœces discovered in any part of the intestines—the liver shewed no strong marks of disease or inflammation, nor was it enlarged—the gall-bladder was full and much distended with bile ; finding it could not be emptied by pressure, it was opened, its contents were black as ink, ink, and of a thick consistence like tar; the stain on the hands from it could not be washed off by soap and many waters; the stain was of a deep yellow colour—the biliary ducts were found to be impervious to a probe, &c.

The family of the deceased have never heard him mention the circumstance of his having at any time swallowed such a substance: the chocolate nut must probably have passed into the stomach by some accident, and by its gravity have obtained a lodgment in this receptacle, and have filled it too completely to be removed. It might have remained in this situation for a long space of time and proved inert, whilst the bowels were properly supplied with healthy bile, and discharged their functions in a proper manner; but so soon as the bile became obstructed, the deficiency of this necessary fluid occasioned torpor and spasm of the intestinal canal; under such circumstances this extraneous substance became mischievous and productive of irritation, inflammation, gangrene, &c. Whether this reasoning be correct or otherwise, the nut thus lodged, proved unquestionably the immediate cause of our patient's death. It was too firmly wedged in the appendicle to have been displaced by any means which could safely have been employed; but had its existence in such situation been foreseen, venesection, the warm bath, and a free internal use of some bland oil might probably have afforded the most likely means for relief.—*New Eng. Journal.*

